# Pentalogy of Cantrell: Sonographic Findings and Toyama Classification

**DOI:** 10.7759/cureus.117141

**Published:** 2026-09-29

**Authors:** Jacques Balayla, Yves Benabu

**Affiliations:** 1 Obstetrics and Gynaecology, Lady Davis Institute (LDI), Jewish General Hospital, Montreal, CAN; 2 Radiology, IRM Sud-Ouest, Montreal, CAN; 3 Radiology, Université de Montréal, Montreal, CAN

**Keywords:** antenatal ultrasound, congenital cardiovascular anomaly, congenital gut anomaly, pentalogy of cantrell, therapeutic termination of pregnancy

## Abstract

A 39-year-old primiparous patient was referred for detailed ultrasonography at 13 weeks and four days of gestation. Examination demonstrated a large midline supraumbilical anterior abdominal wall defect with herniation of abdominal contents, consistent with an omphalocele. The fetal heart was partially located outside the thorax, consistent with ectopia cordis, and an inferior sternal defect was identified. Color and power Doppler confirmed vascular flow within the extracorporeal cardiac structures and their continuity with the fetal circulation. During real-time examination, abnormal intracardiac Doppler flow was observed, and a ventricular septal defect was identified. Deficiency of the anterior diaphragm and diaphragmatic pericardium was suspected but could not be definitively confirmed prenatally. The combination of omphalocele, ectopia cordis, an inferior sternal defect, a ventricular septal defect, and suspected diaphragmatic and pericardial abnormalities was considered most consistent with incomplete Pentalogy of Cantrell. Alternative diagnoses were considered less likely because the defect was midline and supraumbilical, the umbilical cord appeared normal, the fetal axis was relatively preserved, and no limb abnormalities or severe scoliosis were present. Following multidisciplinary counseling, the parents elected termination of pregnancy before genetic testing was undertaken. No post-procedural anatomic examination was available; therefore, pathologic and genetic confirmation was not obtained. This case illustrates that incomplete Pentalogy of Cantrell can be recognized near the end of the first trimester and that Doppler imaging can assist in characterizing associated cardiovascular abnormalities and supporting early multidisciplinary counseling.

## Introduction

Pentalogy of Cantrell is a rare congenital syndrome involving the ventral midline, first described by Cantrell et al. in 1958 [1]. The syndrome classically comprises five structural abnormalities: a midline supraumbilical abdominal wall defect, an inferior sternal defect, a deficiency of the anterior diaphragm, a defect of the diaphragmatic pericardium, and an intracardiac anomaly. Toyama subsequently classified the condition according to the completeness of the pentad, distinguishing complete expression from probable and incomplete forms involving fewer components [2]. The condition has been attributed to abnormal development of the lateral mesoderm during early embryogenesis, resulting in disruption of a shared ventral midline developmental field [3]. Its reported prevalence ranges from approximately one in 65,000 to one in 200,000 live births [4]. Although the extent and severity of the associated abnormalities vary considerably, the coexistence of a supraumbilical omphalocele and ectopia cordis represents a characteristic prenatal imaging pattern [4]. Pentalogy of Cantrell has also been reported in association with chromosomal abnormalities and other congenital anomalies, making consideration of genetic evaluation an important component of prenatal assessment and counseling. Recognition during the first trimester is clinically important because it permits earlier characterization of associated abnormalities, consideration of genetic testing, multidisciplinary consultation, prognostic counseling, and informed discussion of pregnancy-management options. Advances in prenatal ultrasonography allow the characteristic combination of abnormalities to be recognized during the first trimester. Color Doppler can help confirm the extracorporeal location of the fetal heart and assist in evaluating associated intracardiac abnormalities [5]. We report a case of incomplete Pentalogy of Cantrell identified at 13 weeks and four days of gestation using two-dimensional ultrasonography and color and power Doppler imaging.

## Case presentation

A 39-year-old primiparous patient with no relevant medical history or prenatal exposures was referred for a detailed obstetric ultrasound examination at 13 weeks and four days of gestation. Two-dimensional grayscale and Doppler imaging demonstrated that the fetal heart was partially located outside the thorax, consistent with ectopia cordis, and an inferior sternal defect was identified. Color and power Doppler imaging confirmed vascular flow within the extracorporeal cardiac structures and their continuity with the fetal circulation (Figure 1). During real-time examination, abnormal intracardiac Doppler flow was observed, and a ventricular septal defect was identified. No additional intracardiac structural abnormality could be characterized with sufficient certainty at this gestational age. Furthermore, a large midline supraumbilical anterior abdominal wall defect with herniation of abdominal contents, consistent with an omphalocele (Figure 2). The defect was distinct from physiologic midgut herniation expected earlier in gestation because of its size, location, and persistence at this gestational age. Deficiency of the anterior diaphragm and diaphragmatic pericardium was suspected on prenatal imaging but could not be definitively established. The directly visualized combination of a midline supraumbilical omphalocele, ectopia cordis, an inferior sternal defect, and a ventricular septal defect, together with the suspected diaphragmatic and pericardial abnormalities, was considered most consistent with incomplete Pentalogy of Cantrell according to the spectrum described by Toyama [2]. The case was not considered a definitively confirmed complete pentad because two of the five components remained suspected rather than demonstrated, and parents declined further investigations. The principal differential diagnoses included limb-body wall complex, body stalk anomaly, and amniotic band sequence. These possibilities were considered less likely because the defect was midline and supraumbilical, the umbilical cord appeared normal, the fetal axis was relatively preserved, and there were no associated limb abnormalities or severe scoliosis. Following multidisciplinary review, the patient and her partner received counseling regarding the prenatal findings, guarded fetal prognosis, available pregnancy-management options, and genetic evaluation. Genetic testing was discussed/recommended; however, the parents elected termination of pregnancy before testing was undertaken. Dilation and suction curettage were performed without complications. No post-procedural fetal anatomic examination was available to confirm the suspected diaphragmatic and diaphragmatic-pericardial defects. Consequently, neither pathologic nor genetic confirmation of the prenatal diagnosis was obtained. The patient recovered uneventfully and remained clinically well following the procedure.

**Figure 1:**
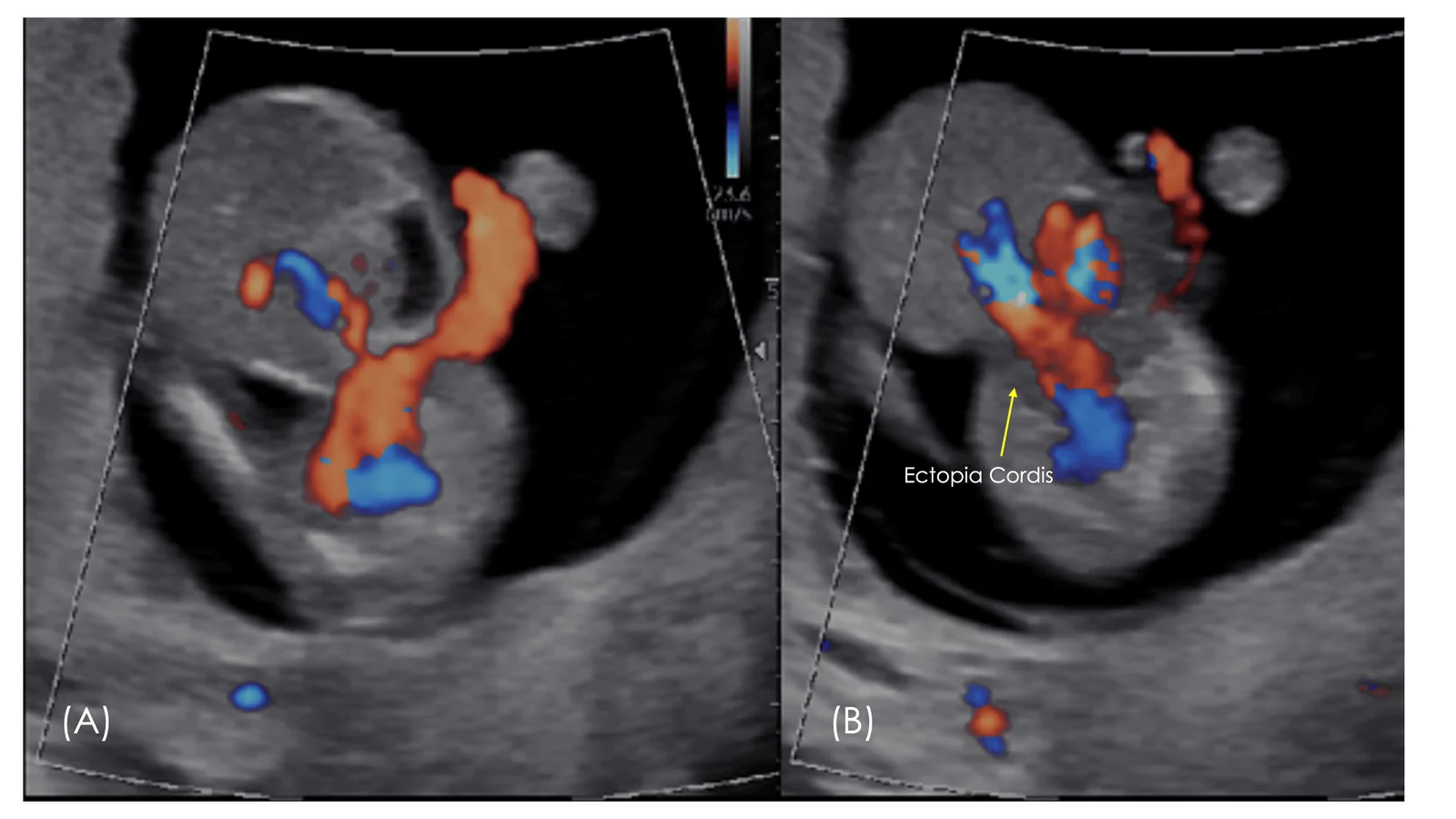
Prenatal ultrasound at 13 weeks and four days of gestation - Pentalogy of Cantrell - ectopia cordis Color Doppler images obtained during the first-trimester examination. (A) Color Doppler demonstrates cardiovascular flow in relation to the anterior thoracoabdominal defect. (B) Color Doppler demonstrates vascular flow within the partially extracorporeal cardiac structures, supporting the diagnosis of ectopia cordis (arrow). During the real-time ultrasound examination, abnormal intracardiac Doppler flow and a ventricular septal defect were additionally identified; these findings are not independently demonstrated by the selected static images.

**Figure 2:**
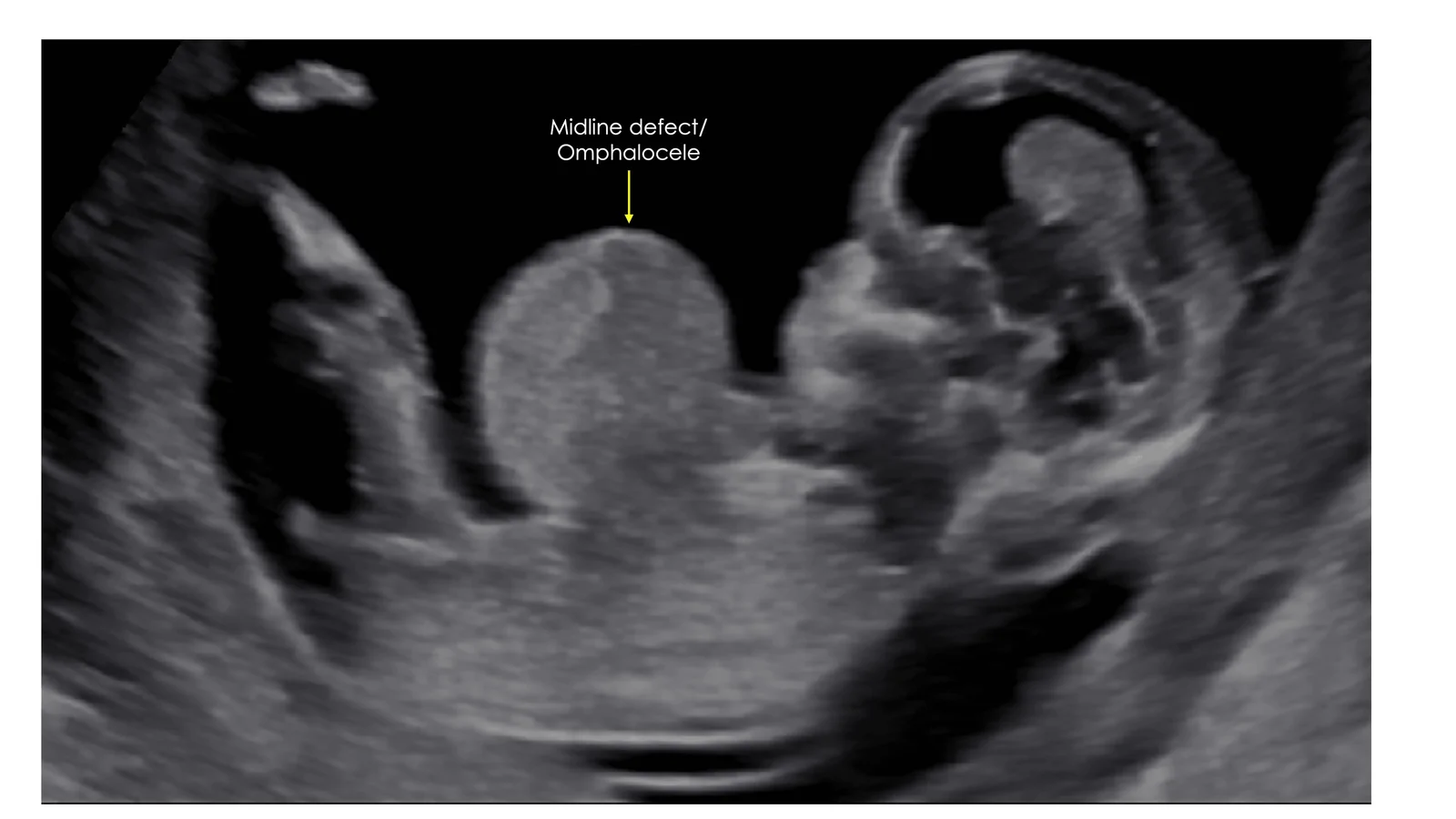
Prenatal ultrasound at 13 weeks and four days of gestation - Pentalogy of Cantrell - midline defect Two-dimensional grayscale ultrasonography at 13 weeks and four days of gestation demonstrating a large midline supraumbilical anterior abdominal wall defect with herniation of abdominal contents, consistent with an omphalocele (arrow).

## Discussion

The present case demonstrates prenatal recognition of probable/incomplete Pentalogy of Cantrell near the end of the first trimester. The simultaneous visualization of a midline supraumbilical omphalocele and ectopia cordis provided the principal diagnostic pattern. Identification of an inferior sternal defect and ventricular septal defect provided additional components of the syndrome, while deficiencies of the anterior diaphragm and diaphragmatic pericardium were suspected but could not be definitively confirmed. This distinction is important when applying the classification proposed by Toyama [2]. The classical complete form requires demonstration of all five components of the pentad. In the present case, four structural components were identified or strongly supported prenatally-the supraumbilical abdominal wall defect, inferior sternal defect, ectopia cordis associated with the thoracoabdominal defect, and an intracardiac abnormality-while the anterior diaphragmatic and diaphragmatic-pericardial abnormalities remained suspected. Accordingly, the findings are more appropriately described as probable/incomplete Pentalogy of Cantrell rather than a definitively confirmed complete pentad. Because pregnancy termination occurred before genetic testing and no post-procedural anatomic examination was available, definitive pathologic or genetic confirmation could not be obtained. Several severe fetal disorders can produce abnormalities of the anterior abdominal wall and apparent exteriorization of thoracic or abdominal organs. Limb-body wall complex, body stalk anomaly, and amniotic band sequence are therefore important differential considerations [6]. Limb-body wall complex and body stalk anomaly are commonly associated with marked distortion of the fetal axis, severe scoliosis, limb abnormalities, or an absent or markedly shortened umbilical cord. Amniotic band sequence generally produces asymmetric and irregular defects, often accompanied by limb disruption. In the present case, the relatively preserved fetal axis, normal appearance of the umbilical cord, absence of limb abnormalities or severe scoliosis, and midline supraumbilical location of the defect favored Pentalogy of Cantrell. Color Doppler imaging was useful in supporting the diagnosis. Demonstration of cardiovascular flow within structures partially exteriorized from the fetal thorax supported the presence of ectopia cordis rather than an extracardiac soft-tissue structure. During real-time examination, Doppler imaging also demonstrated abnormal intracardiac flow, and a ventricular septal defect was identified. Importantly, the examination did not permit sufficiently detailed characterization of additional intracardiac structural abnormalities; therefore, the nonspecific designation of a “complex cardiac malformation” was avoided. Previous first-trimester reports have similarly emphasized the value of Doppler imaging for confirming ectopia cordis and defining its relationship to the omphalocele and thoracic structures [5]. The prognosis of Pentalogy of Cantrell is determined principally by the severity of the cardiac abnormality and the extent of thoracoabdominal involvement [7]. Complete ectopia cordis with an exposed heart is associated with an especially poor likelihood of prolonged survival. Associated intracardiac abnormalities may further complicate postnatal stabilization and surgical management. Accurate prenatal characterization is therefore important not only for establishing the likely diagnosis but also for counseling regarding anticipated outcomes and available pregnancy-management options. Genetic evaluation is also relevant when Pentalogy of Cantrell is suspected prenatally because chromosomal and other congenital abnormalities have been described in association with the syndrome. In the present case, genetic testing was discussed/recommended as part of prenatal counseling but was not undertaken because the parents elected termination before testing. Consequently, no conclusion regarding an underlying genetic abnormality can be drawn from this case. The present report has several limitations. Most importantly, the anterior diaphragmatic and diaphragmatic-pericardial defects were suspected but not definitively demonstrated. In addition, no post-procedural anatomic examination was available, preventing confirmation of these components and definitive classification of the pentad. Genetic testing was not performed. Finally, detailed fetal echocardiographic characterization at this early gestational age was limited, and the ventricular septal defect was the only specific intracardiac structural abnormality identified. These limitations support describing the prenatal diagnosis as probable/incomplete Pentalogy of Cantrell. Nevertheless, the combination of a midline supraumbilical omphalocele, ectopia cordis, an inferior sternal abnormality, and an intracardiac defect constitutes a highly suggestive prenatal pattern. Recognition at 13 weeks and four days allowed early multidisciplinary consultation and informed counseling regarding prognosis, further diagnostic evaluation, and pregnancy-management options.

## Conclusions

Probable/incomplete Pentalogy of Cantrell can be recognized near the end of the first trimester when ultrasonography demonstrates the characteristic combination of a midline supraumbilical omphalocele and ectopia cordis together with additional ventral midline or intracardiac abnormalities. Careful assessment should distinguish directly visualized components from abnormalities that are only suspected, particularly when definitive postnatal or pathologic confirmation is unavailable. Color and power Doppler imaging can help confirm the extracorporeal cardiac position and assess associated cardiovascular abnormalities. In this case, the imaging findings strongly supported probable/incomplete Pentalogy of Cantrell, although suspected diaphragmatic and pericardial defects could not be confirmed, and neither pathologic nor genetic confirmation was obtained. Early recognition facilitates timely multidisciplinary assessment, consideration of genetic evaluation, prognostic counseling, and informed decision-making regarding pregnancy management.

## References

[1] Fazea M, Alhameli M, Ahmed F, Askarpour MR, Murshed W, Jarwsh A, Alkbous A (2022). Pentalogy of Cantrell associated with ectopia cordis: a case report. Pediatric Health Med Ther.

[2] Toyama WM (1972). Combined congenital defects of the anterior abdominal wall, sternum, diaphragm, pericardium, and heart: a case report and review of the syndrome. Pediatrics.

[3] Carmi R, Boughman JA (1992). Pentalogy of Cantrell and associated midline anomalies: a possible ventral midline developmental field. Am J Med Genet.

[4] Williams AP, Marayati R, Beierle EA (2019). Pentalogy of Cantrell. Semin Pediatr Surg.

[5] Türkçapar AF, Sargın Oruc A, Öksüzoglu A, Danışman N (2015). Diagnosis of pentalogy of cantrell in the first trimester using transvaginal sonography and color Doppler. Case Rep Obstet Gynecol.

[6] Cakiroglu Y, Doger E, Yildirim Kopuk S, Babaoglu K, Caliskan E, Yucesoy G (2014). Prenatal diagnosis of Cantrell’s pentalogy associated with agenesis of left limb in a twin pregnancy. Case Rep Obstet Gynecol.

[7] van Hoorn JH, Moonen RM, Huysentruyt CJ, van Heurn LW, Offermans JP, Mulder AL (2008). Pentalogy of Cantrell: two patients and a review to determine prognostic factors for optimal approach. Eur J Pediatr.

